# Effect of Mucopolysaccharide Polysulfate on Sweating Function and Skin Dryness in a Mouse Model of Streptozotocin-Induced Diabetes

**DOI:** 10.1159/spp/aetag005

**Published:** 2026-10-10

**Authors:** Wataru Sugimoto, Hironobu Ishimaru, Sanae Hirota, Ryo Tadakuma, Ryo Aikawa, Rina Furumoto, Takaaki Doi, Yuhki Ueda

**Affiliations:** Drug Development Research Laboratories, Kyoto R&D Center, Maruho Co., Ltd., Kyoto, Japan

**Keywords:** Asteatosis, Diabetes mellitus, Streptozotocin, Mucopolysaccharide polysulfate, Sweating dysfunction

## Abstract

**Introduction:**

Asteatosis is characterized by reduced water content in the stratum corneum. Patients with diabetes have been reported to experience reduced sweating, potentially leading to asteatosis. A common treatment approach for asteatosis is the use of moisturizers, such as mucopolysaccharide polysulfate (MPS) cream. However, the therapeutic mechanism of MPS in diabetes-induced asteatosis remains unknown. In this study, we investigated the role of sweating in the pathophysiology of asteatosis in diabetes and to confirm the therapeutic effect of MPS cream using a streptozotocin (STZ)-induced diabetic mouse model (STZ-DM mice).

**Methods:**

STZ-DM mice were generated by administering STZ to C57BL/6JJcl mice. Approximately 2 weeks after STZ administration, sweating function and skin surface hydration in the footpads, where sweat glands are located, were evaluated, with or without topical occlusive application of moisturizers including MPS cream for more than 10 days.

**Results:**

After STZ treatment, sweating function in STZ-DM mice gradually decreased from week 3 to 9. Furthermore, skin surface hydration and sweating function decreased over time. Topical application of MPS cream alleviated diminished sweating and skin surface hydration in STZ-DM mice. Moreover, pilocarpine-induced sweating response was attenuated in STZ-DM mice, indicating that impairment of sweating function in these mice involves neuropathic dysfunction. Topical application of MPS cream improved the pilocarpine-induced sweating response.

**Conclusion:**

The effects of MPS on the increasing water content of SC in STZ-DM mice may be partly mediated by sweating. This is the first study to demonstrate that the ameliorative effect of MPS on diabetic dry skin is mediated by enhanced sweating via neuropathy inhibition.

## Introduction

Asteatosis, also referred to as xerosis, causes dry skin accompanied by physiological changes, such as reduced water content in the stratum corneum (SC), which may lead to disruption of the skin barrier function [1]. Asteatosis development has various factors, such as physiological (e.g., age) and environmental (e.g., climate change) factors. These also include nonphysiological factors, such as atopic dermatitis, psoriasis vulgaris, and other skin diseases as well as diabetes mellitus and other systemic diseases.

The SC, which is the outermost layer of the skin, acts as a barrier against the external environment. The water content in the SC is maintained at a certain level to preserve its barrier function. Recent studies have shown that sweating plays an important role in maintaining the water content in the SC [2, 3]. Sweat is produced from plasma in the sweat glands and discharged onto the skin via the sweat ducts. Sweat is the primary source of water for the SC. When sweating is impaired, the resulting reduced water supply to the SC decreases its moisture content, potentially contributing to asteatosis development [4].

Asteatosis often occurs in patients with diabetes mellitus and is one of the reasons known to cause several complications, such as skin infections and diabetic foot lesions, as the reduced water content associated with asteatosis can result in skin fissures and impaired skin barrier function [5–7]. Various studies on the pathophysiology of asteatosis in patients with diabetes have been conducted, and reduced sweating is thought to contribute to skin dryness [5, 8]. Moreover, many studies have demonstrated that patients with diabetes mellitus experience reduced sweating owing to autonomic neuropathy [9, 10]. This reduction is considered to be a cause of asteatosis among diabetes patients.

Generally, moisturizers are used to treat asteatosis. Mucopolysaccharide polysulfate (MPS) cream (MPS-C) is a typical moisturizer used to treat asteatosis in Japan. MPS not only helps maintain the water content in the SC but also exerts diverse pharmacological effects [11], including promotion of sweating via blood flow promotion [12]. Although MPS-C is used to treat asteatosis in patients with diabetes mellitus, the underlying pharmacological mechanism for its therapeutic efficacy against diabetes mellitus-induced asteatosis is unclear.

This study aimed to elucidate the involvement of sweating in the pathophysiology of diabetes mellitus-induced asteatosis and the pharmacological mechanism of MPS for asteatosis. First, we used a streptozotocin (STZ)-induced diabetic mouse model (STZ-DM mice) to determine the association between reduced sweating and the SC water content in diabetes mellitus. Sweating function and skin surface hydration (SSH) in the footpad, where sweat glands are located in mice, decreased over time as the blood glucose levels increased, as previously known in humans. We further evaluated the efficacy of MPS-C in the same mouse model and demonstrated that MPS-C has sweating maintenance effect by preserving autonomic neural function in STZ-DM mice. This was the first study to demonstrate that the efficacy of MPS against diabetic asteatosis was mediated by the promotion of sweating.

## Methods

### Mice and Housing Conditions

Seven-week-old male C57BL/6JJcl mice were purchased from CLEA Japan, Inc. (Tokyo, Japan). All mice were housed one animal per cage and acclimated for at least 5 days before use. Mice were maintained under specific pathogen-free conditions on a 12-h light/dark cycle (lights on at 7:00 and off at 19:00) and were provided a laboratory diet (CRF-1, Oriental Yeast Co., Ltd., Tokyo, Japan) and ultraviolet-sterilized water ad libitum at Maruho Co., Ltd.’s Kyoto R&D Center animal facility. The protocols of this study were approved by the Animal Care Committee of Maruho Co., Ltd.

### STZ-Induced Diabetic Model Mice

Mice were assigned to a naïve control group and an STZ (Sigma, MO, US; 70 mg/kg i.p.) administration group by 10 weeks old. The STZ group with 10 weeks old mice received repeated administrations of STZ solution over 5 consecutive days, with the first day of administration designated as Day 1. STZ solution was prepared immediately prior to injection by dissolving STZ in 50 mM citrate buffer (pH 4.5). After 2 weeks, blood glucose levels were measured using LAB Gluco (ForaCare Japan Co., Ltd., Tokyo, Japan), and animals exhibiting blood glucose concentrations exceeding 400 mg/dL were utilized as STZ-DM mice. If blood glucose levels exceeded the upper limit, 600 mg/dL, the values were capped at 600 mg/dL.

### Measurement of SSH and Sweating Response

These parameters were measured in the right hind paws of mice. Measurement of sweating responses using impression mold technique (IMT) included minor modifications of previously published methods [4, 13]. Sweat droplet impressions were obtained from the murine footpads using Silasoft^®^ (DETAX GmbH & Co. KG), a silicone-based impression material. After application to the footpad, the material was allowed to harden completely, thereby creating a precise negative cast of the sweat droplets present on the skin surface. In this IMT, the impressions of the sweat droplets were counted using a dissection microscope and quantified via their number [4]. Usually, impression molds hardened in 2–3 min, and they were then removed. Sweat droplet impressions on the mold were counted using a dissection microscope and distal 4-paw pads of whole footpad (6-paw pads) were counted.

SSH was measured using a Skicon-200EX (Yayoi Co., Ltd., Tokyo, Japan) as described previously [14]. First, mice were restrained in a holder without anesthesia, with their right hindlimb extended. This device evaluates SSH by measuring the conductivity of the skin surface when the probe is applied to the right footpad, including the distal 4-paw pads.

### Topical Moisturizer Treatment

These moisturizers were applied to the right hind paws of mice. Two independent studies were conducted on day 17 using diabetic mice: the first study grouped the mice into the MPS-C (Hirudoid^®^ cream 0.3%, Maruho Co., Ltd., Osaka, Japan) group or MPS-C placebo (MPS-P, Maruho Co., Ltd.) group, and the second study grouped mice into the MPS-C, white petrolatum (WP) (PROPETO^®^, Maruishi Pharmaceutical Co., Ltd., Tokyo, Japan), urea cream (UC) (Pastaron^®^ cream 10%, Sato Pharmaceutical Co., Ltd., Tokyo, Japan) treatment groups, or the non-treatment group. MPS-C, MPS-P, WP, and UC were applied to the footpads (40 µL per footpad, twice daily on weekdays and once daily on weekends) with occlusion for over 10 days; then we measured sweating function and SSH. The topical agents were administered using 1 mL tuberculin syringes (Terumo Co., Ltd.) and the administered volume was controlled by using the graduations on the syringe. For occlusion, the footpad was first covered with a cotton pad (4 × 2 cm diamond). This was secured with non-stretch tape (Daiso Industries Co., Ltd.), ensuring the adhesive side did not touch the skin. The area was then further wrapped with an elastic air-through bandage (Elastopore No. 50, Nichiban Co., Ltd.) up to the ankle, which was folded back to prevent its adhesive from touching exposed skin.

### Measurement of Sweating Response after Pharmacological Stimulation

Responsiveness to pharmacologic stimulation in diabetic mice was assessed with pilocarpine using IMT. Mice were anesthetized using 2%–3% isoflurane (Viatris Inc, PA, US) and pre-administration measurement was held by IMT. Pilocarpine (2.5 mg/kg; Nacalai tesque, Kyoto, Japan), dissolved in distilled water, was injected subcutaneously into the back skin and IMT was performed within 20 min after pilocarpine administration.

### Immunohistochemistry

The skin tissue from the mice footpad was stained using the immunohistochemistry method. The quantitative analysis was performed using the IMARIS software (Oxford Instruments, Northern Ireland, UK). Additional details are included in the online supplementary Materials and Methods (for all online suppl. material, see https://doi.org/10.1159/spp/aetag005).

### Statistical Analysis

The data are presented as the mean ± standard error. Statistical analysis was performed using the EXSUS software (Ver.10.1) (CAC EXIXARE Corporation, Tokyo, Japan). Differences between two groups were analyzed using the Student’s *t* test or Aspin-Welch’s *t* test, following an *F* test. Comparisons among multiple groups were analyzed using Dunnett’s test. Statistical significance was indicated by *p* < 0.05.

## Results

Sweating function in STZ-DM mice is impaired depending on the period of hyperglycemia, and the SSH is reduced due to the impairment in sweating function. First, we determined whether sweating function impairment and reduced water content in the SC, which have been observed in patients with diabetes mellitus, occur in diabetic mouse models as well. Thus, we developed an STZ-DM mouse model, a representative model of type 1 diabetes mellitus, and measured sequentially the number of sweat droplets and SSH among STZ-DM mice (Fig. 1a). At 2 weeks after STZ administration (week 3), the blood glucose levels in the STZ-DM mice were significantly higher than those in the naïve group and remained elevated until 8 weeks following STZ administration (week 9) (Fig. 1b). Compared with naïve mice, the number of sweat droplets in STZ-DM mice began to decline at week 3 and showed a significant reduction from week 5 onward (Fig. 1c). Similarly, SSH in STZ-DM mice was significantly reduced starting at week 3 (Fig. 1d). The number of sweat droplets and SSH decreased in the STZ-DM mice until week 9, whereas both indices remained unchanged in the naïve mice.

**Fig. 1.**
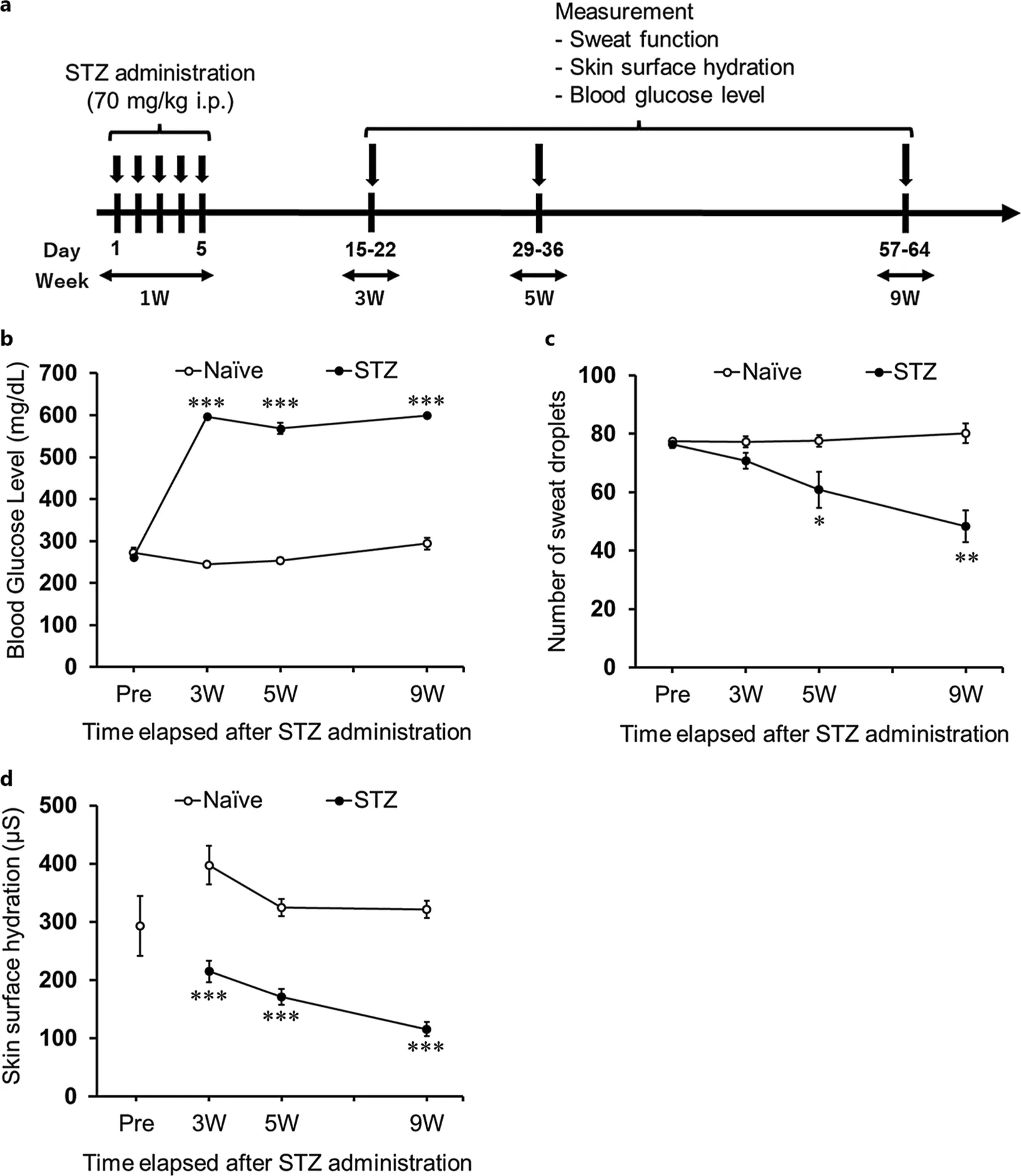
Sweating function and SSH at the footpad decreased over time in STZ-DM mice. **a** Experimental scheme. The changes of blood glucose levels (**b**), number of sweat droplets (**c**) and SSH (**d**). Each chart represents the mean ± standard error. The number of samples in each group is as follows. **b**, **c** naïve: *n* = 5. **d** Pre: *n* = 5, 4 (3W–9W). **b**, **c** STZ: *n* = 11. **d** Pre: *n* = NA, 11 (3W–9W). *p < 0.05; **p < 0.01; ***p < 0.001 by the Student’s or Aspin-Welch’s *t* test compared with the naïve. SSH, skin surface hydration; NA, not assessed.

### Topical Application of MPS Cream to STZ-DM Mice Alleviated the Impairment in Sweating Function and the Decreased SSH

MPS-C has been reported to promote sweating and increase the water content in the SC [12, 15]. Therefore, we evaluated the effects of repeated MPS-C application to the footpad of STZ-DM mice on the sweating function impairment and the decreased SSH (Fig. 2a). Thus, the MPS-C group had a higher number of sweat droplets and SSH than the MPS placebo (MPS-P) group (Fig. 2b, c). Furthermore, to compare the effects of MPS-C and other moisturizers used for diabetes mellitus-induced dry skin, MPS-C, WP, and UC were topically applied to the footpad of STZ-DM mice, and the number of sweat droplets and the SSH were measured. MPS-C tended to increase the number of sweat droplets compared with non-treatment STZ-DM mice (*p* = 0.12), whereas WP and UC did not (Fig. 2d). The SSH did not differ between the MPS-C group and the WP or UC group (Fig. 2e).

**Fig. 2.**
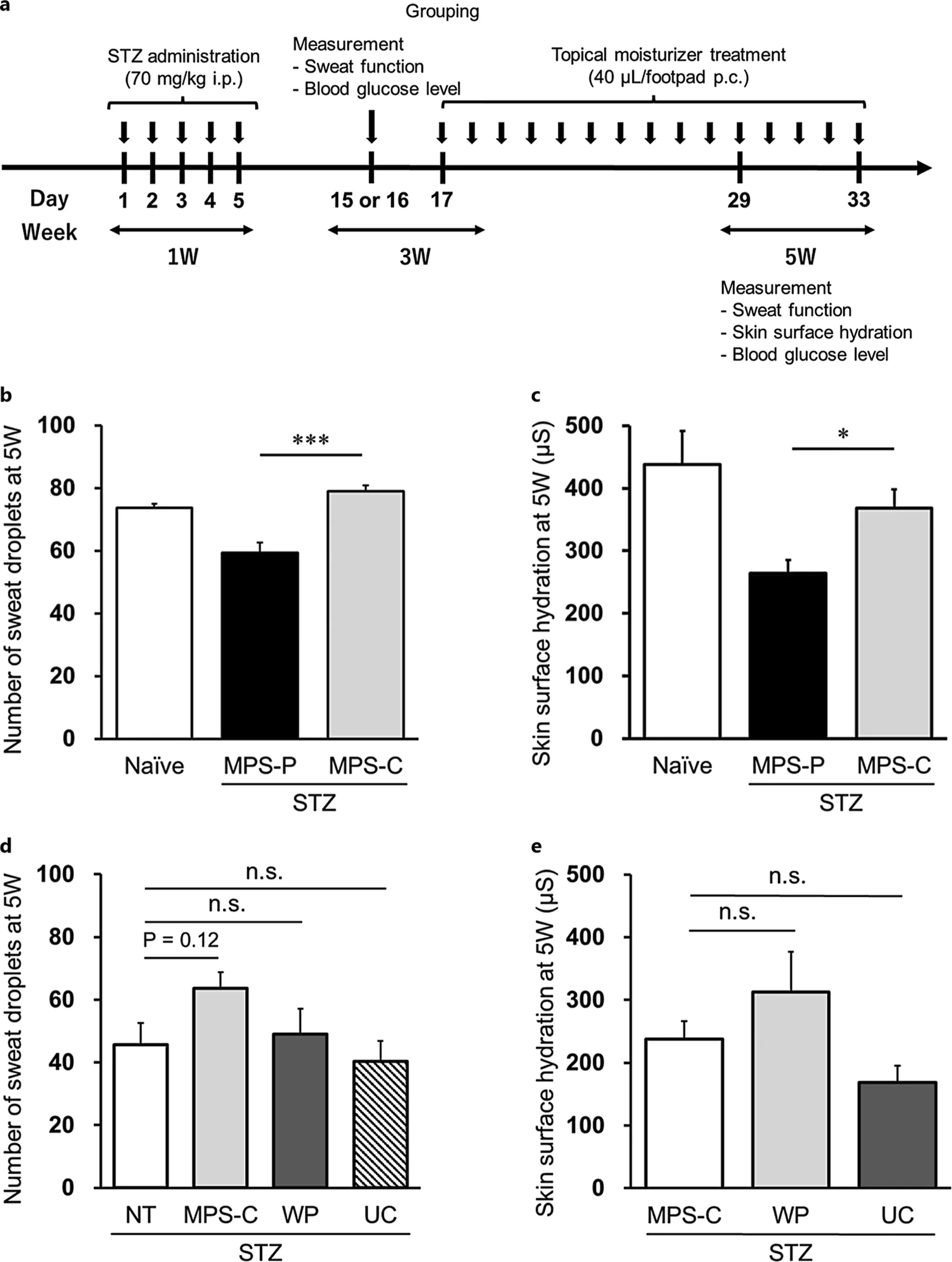
MPS-C treatment alleviated the impairment of sweating function and SSH in STZ-DM mice. **a** Experimental scheme. Number of sweat droplets (**b**) and SSH (**c**) in STZ-DM mice at the footpad after topical application of MPS-C or MPS-P at week 5. Number of sweat droplets (**d**) and SSH (**e**) in STZ-DM mice at the footpad after treatment of each topical application. Each column represents the mean ± standard error. The number of samples in each group is as follows. **b**, **c** Naïve: *n* = 4; MPS-P and MPS-C: *n* = 7. **d** NT: *n* = 10, MPS-C: *n* = 10, WP: *n* = 7, UC: *n* = 7. **e** All groups: *n* = 7. *p < 0.05 and **p < 0.01 by the Student’s or Aspin-Welch’s *t* test compared with the respective control. ns, not significant; SSH, skin surface hydration; NT, non-treatment.

### Densities of Blood Vessels and Peripheral Nerve Fibers around the Sweat Glands in the Footpad of STZ-DM Mice Did Not Change

Previous studies have reported changes in vascular architecture and reduced peripheral nerve density in patients with diabetes mellitus [10, 16]. We herein determined whether these changes also contribute to sweating function impairment in STZ-DM mice. Footpad skin samples of mice were collected to measure the densities of the blood vessels and nerve fibers around the sweat glands. We built 3D models of the regions around the sweat glands and compared the volume ratios of blood vessels (CD31) and nerve fibers (Tuj1) using immunohistochemistry staining images (Fig. 3a, b). The process used to construct the 3D models is shown in the online supplementary figures (Fig. S1 a, b). Thus, the densities of blood vessels and nerve fibers around the sweat glands did not differ between naïve and STZ-DM mice, even at week 9 (Fig. 3a, b). These results suggest that the reduced sweating function observed in STZ-DM mice in this study is not attributable to structural damage of blood vessels or nerve fibers.

**Fig. 3.**
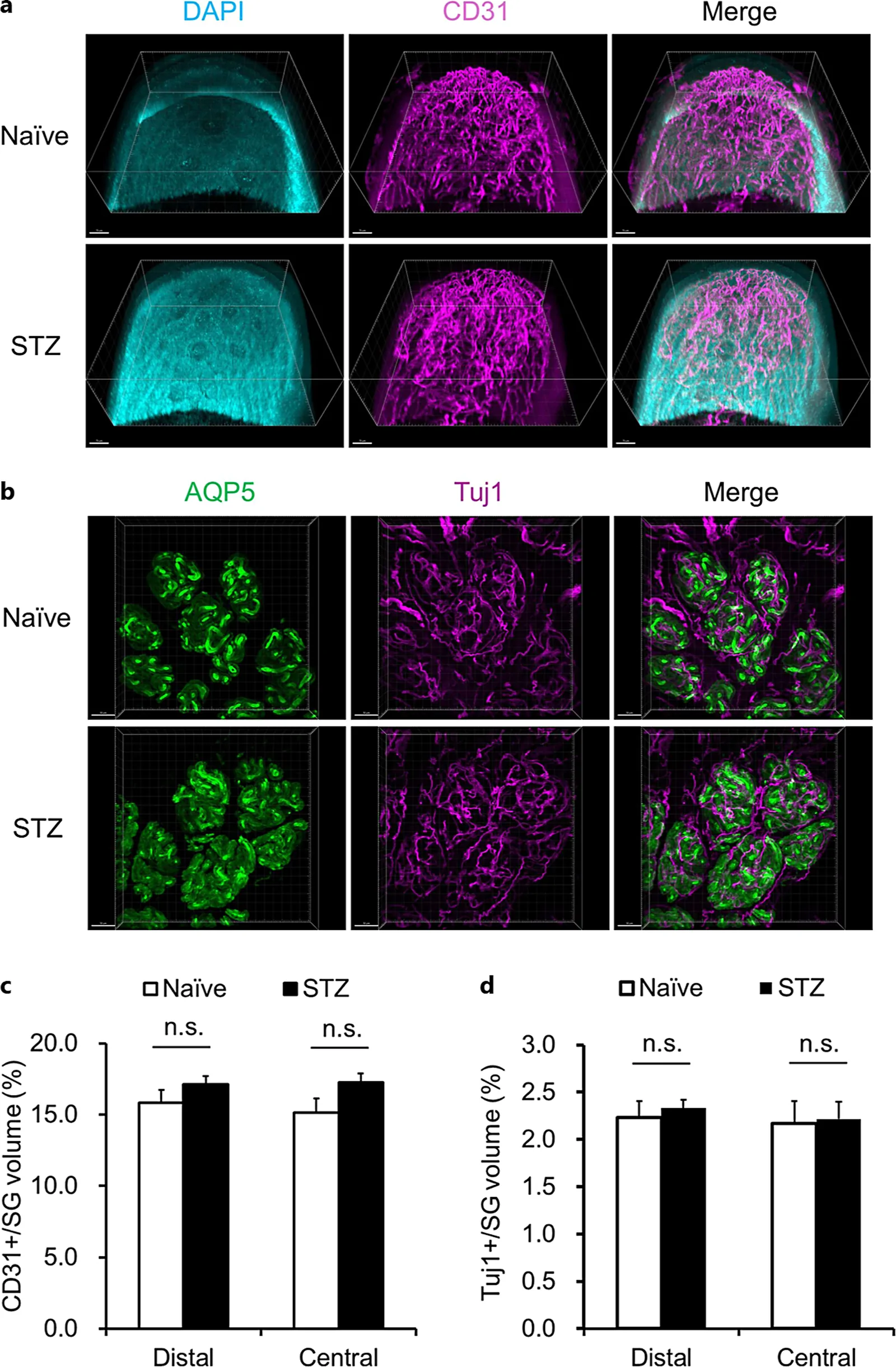
There was no change in the density of blood vessels or nerve fibers around sweat glands in STZ-DM mice. Representative immunofluorescent images illustrating histological changes in the footpad skin of naïve (top panels) and STZ-DM (bottom panels) mice at week 9. **a** Vascular structures in the dermis of the footpad skin. Blood vessels were visualized by staining for CD31 (magenta). Nuclei were counterstained with DAPI (blue). Individual channels (DAPI and CD31) and the merged image are shown from left to right. **b** Nerve fibers surrounding the eccrine sweat glands. Nerve fibers were identified by staining for the neuronal marker Tuj1 (magenta). Sweat gland epithelial cells were stained for AQP5 (green). Individual channels (AQP5 and Tuj1) and the merged image are shown from left to right. The density of blood vessels (**c**) and nerve fibers (**d**) around sweat gland of mice at week 9. Each column represents the mean ± standard error. The number of samples in each group is as follows. **c** Naïve: *n* = 5; STZ: *n* = 6–7. **d** Naïve: *n* = 4; STZ: *n* = 8, using Student’s or Aspin-Welch’s *t* test compared with the naïve. ns, not significant; distal, the paw pad located at toe side of footpad; central, the paw pad located at central part of footpad.

### The Response to Sweat Induction by a Cholinergic Agonist Was Attenuated in STZ-DM Mice

It has been reported that autonomic neuropathy is involved in impairment of sweating function in patients with diabetes mellitus [9, 10]. As aforementioned, the STZ-DM mice used in this study showed no structural changes in nerve fibers. Therefore, we focused on autonomic nerve functional impairment. To elucidate the involvement of autonomic neuropathy in sweating function impairment in STZ-DM mice, we evaluated the sweating response to the cholinergic agonist pilocarpine. We measured the number of sweat droplets following subcutaneous administration of pilocarpine to the back skin of mice under inhalation anesthesia. In naïve mice, the number of sweat droplets decreased under inhalation anesthesia and increased following pilocarpine administration (Fig. 4a). In evaluations of pilocarpine-induced sweating responses at week 5 and week 9, sweating responses in STZ-DM mice were significantly diminished compared with those in naïve mice. Furthermore, the sweating response in STZ-DM mice exhibited a progressive decline over time (Fig. 4b). This suggests that autonomic neuropathy is involved in the previously observed sweating function impairment in STZ-DM mice. The same evaluation was conducted following topical application of MPS-C in STZ-DM mice. The number of sweat droplets after pilocarpine administration was measured at week 5, as illustrated in Figure 2a. The MPS-C group showed a higher number of sweat droplets than the MPS-P group (Fig. 4c). These results suggest that autonomic peripheral neuropathy may be ameliorated by topical application of MPS-C.

**Fig. 4.**
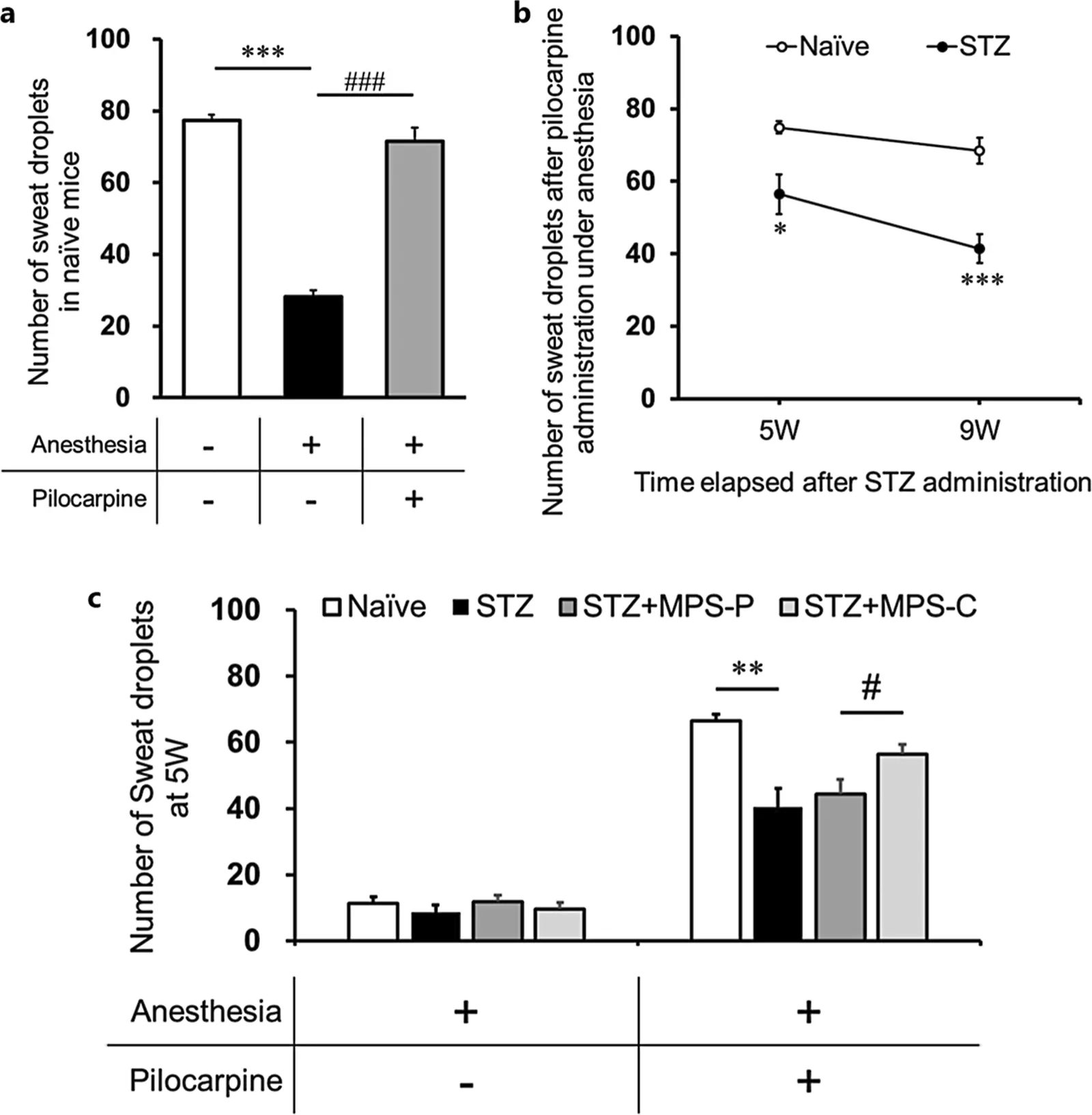
MPS-C improved the diminished sweating response to the cholinergic agonist pilocarpine in STZ-DM mice. **a** Number of sweat droplets under anesthesia and after administration of pilocarpine in naïve mice. **b** Change in the number of sweat droplets after administration of pilocarpine in STZ-DM mice. **c** Number of sweat droplets after administration of pilocarpine following topical treatments of MPS-C and MPS-P at week 5. Each column and chart represent the mean ± standard error. The number of samples in each group is as follows. **a***n* = 5. **b** Naïve: *n* = 4, 5; STZ: *n* = 7, 8. **c** Naïve: *n* = 8; STZ: *n* = 10; MPS-P and MPS-C: *n* = 12. **a** ***p < 0.001 by using Student’s or Aspin-Welch’s *t* test compared with the naïve. ###*p* < 0.001 by using Student’s or Aspin-Welch’s *t* test compared with the naïve under anesthesia. **b** *p < 0.05 and ***p < 0.001 by using Student’s or Aspin-Welch’s *t* test compared with the naïve. **c** *p < 0.05 and **p < 0.01 by using Student’s or Aspin-Welch’s *t* test compared with the naïve. #*p* < 0.05 by using Student’s *t* test compared with the MPS-P.

## Discussion

We investigated the therapeutic effects of MPS-C in diabetic mouse models, focusing on the moisturizing and sweat-promoting effects of MPS-C. MPS-C prevented sweating function impairment and decrease in SSH, which occurred in STZ-DM mice. It has been reported that patients with diabetes mellitus exhibit symptoms of asteatosis, typically presenting with skin dryness and diminished sweating [5, 17, 18]. Diminished sweating in humans has been reported to be one of the causes of reduction in the SC water content [2, 17, 19], and decreased sweating in patients with diabetes mellitus may cause dry skin. This is also the case in mice. The sweating function and SSH in the mouse footpad, where the sweat glands are located in mice, showed similar changes over time [20]. In the STZ-DM mice used in this study, the sweating function and SSH also diminished over time as the blood glucose levels increased, indicating the skin dryness in mice with sweating function impairment, similar to patients with diabetes mellitus. The number of sweat droplets and the SSH showed similar longitudinal changes with the amount of decrease increasing over time until week 9. Previous reports suggested that skin dryness may be caused by diminished sweating in patients with diabetes mellitus [5]. Our result also shows that the SSH decreases and diminished sweating in STZ-DM mice; these are similar phenotypes to patients with diabetes mellitus. A significant decline in the number of sweat droplets was observed at week 5, whereas the reduction in SSH was detected as early as week 3. These findings suggest that even a modest decrease in sweating at week 3 may substantially affect SSH.

To elucidate the mechanism of MPS, we topically applied MPS-C to the footpad of STZ-DM mice and found that the sweat decrease was alleviated compared with that in the placebo group. As this effect was not observed with WP or UC, the effect was attributed to MPS, which is the active ingredient in the MPS-C. These results suggest that sweating promotion is a characteristic effect of MPS. MPS has been reported to increase skin blood flow via vasodilation by increasing the NO production from vascular endothelial cells [21]. As sweat is produced in the sweat glands using water from plasma, an increase in blood flow is likely to increase water supply to the sweat glands. The topical application of MPS to the footpad of mice has been reported to promote sweating through increased blood flow [12]. Therefore, the increase in blood flow was considered to have partly contributed to the sweat-promoting effect of MPS-C observed in this study.

MPS-C also prevented the decrease of SSH in STZ-DM mice. Sweat is known to be a source of water and natural moisturizing factors (NMFs), such as lactic acid and urea [3]. Studies have demonstrated that NMFs capture water in the SC and are involved in the maintenance of the water content [3, 22]. Diminished sweating leads to reduction in the SC water content by both reducing the direct water supply and impairing the ability of retaining water due to NMF depletion in the SC [3, 4]. Therefore, water and NMFs supplied through sweating were considered to be involved in the retaining water content in the SC by MPS application. Furthermore, MPS itself has been reported to physically retain water via its structural moieties capable of achieving hydrogen bonding with water molecules in close proximity [15]. Therefore, the ability of MPS to hold water in the surrounding tissues through its own structural moieties and to promote sweating potentially contributes to the prevention of the decrease in SSH in STZ-DM mice.

However, no differences were observed among STZ-DM mice treated with MPS-C, WP, and UC in terms of SSH at week 5. Meanwhile, WP and UC did not show any effect in alleviating diminished sweating. Given that STZ-DM mice exhibit a time-dependent decrease in sweating, prolonged application of MPS-C may be required to demonstrate its effect in maintaining SSH through its sweat-promoting effect. Therefore, further studies on the changes in SSH for longer durations following MPS-C administration are needed.

Sweating is induced by acetylcholine released from sympathetic nerves. Decreased sweating caused by autonomic dysfunction has been reported in patients with both type 1 and type 2 diabetes mellitus [10, 23–27]. In this study, no changes in the density of blood vessels or nerve fibers around sweat glands were observed in the footpad skin of STZ-DM mice. Therefore, we evaluated nerve dysfunction using pilocarpine-induced sweating as a guide. In STZ-DM mice, the sweating response to pilocarpine was attenuated from week 5 onward. In addition, as shown in Figure 1c, the number of sweat droplets in STZ-DM mice began to decrease at week 3. These results suggest that autonomic dysfunction may be potentially involved in diminished sweating observed from week 3, although no changes in the nerve fiber density occurred even at week 9 in the STZ-DM mice used in this study. A previous study has reported a reduction in the density of nerve fibers around sweat glands in db/db mice, which were genetically modified to develop diabetes mellitus [28]. In this study, the STZ-DM mice were expected to demonstrate similar changes; however, such changes were not observed even by week 9. This suggests that the duration of hyperglycemia may not have been long enough or that factors other than hyperglycemia were involved in the decrease in the nerve fiber density. The association between different diabetic mouse models and the decrease in nerve fiber density, including the factors underlying the association, is a point to consider. Moreover, repeated MPS-C administration alleviated the reduced sweating response in STZ-DM mice by the cholinergic agonist, indicating the protective effect of MPS-C against autonomic dysfunction. The MPS-C administration was initiated at week 3, when sweating dysfunction was considered to have begun. Therefore, the reduction of sweating response associated with autonomic dysfunction probably has been prevented by the MPS-C for the administration from an early stage during which the sweating dysfunction was mild. Previous studies have also demonstrated that sweating function impairment starts at the early stages of diabetic neuropathy [24] and that early initiation of therapeutic intervention for autonomic dysfunction is potentially effective.

Protection from autonomic neuropathy in DM mice has been reported to be involved in sweating function preservation [29]. In this study, MPS mitigated sweating function impairment possibly through its protective effect against neuropathy caused by persistent hyperglycemia. Although this mechanism remains to be elucidated, one possibility is the involvement of blood flow promotion by MPS [21]. Various mechanisms are reportedly involved in the pathophysiology of diabetic neuropathy, including impaired blood flow. Particularly, ischemia and hypoxia in microvessels, accompanied by decreases in angiogenic and neurotrophic factors, including vascular endothelial growth factor and nerve growth factor, are considered to cause neuropathy [30]. Topical application of MPS-C to the skin has been reported to reach the dermis [11]. Therefore, the preservation of autonomic nerve function could be attributed to the effects of MPS around sweat glands in the dermis and its ability to promote blood flow, thereby supplying oxygen, angiogenic and neurotrophic factors to the microvessels and peripheral nerves around the sweat glands. Previous studies have demonstrated the therapeutic effects of vasodilating agents [31], ultrasound therapy for blood flow promotion [32], and local delivery of vascular endothelial growth factor [33] on neuropathy. Therefore, the findings suggest that the blood flow promoting effect of MPS treatment for diabetes-induced sweating function impairment may have great clinical importance.

A limitation of this study was that we were unable to evaluate the therapeutic effect of MPS-C on severe sweating dysfunction in STZ-DM mice. The severity of sweating dysfunction in patients with diabetes varies, and there are cases where the dysfunction has already progressed. In this study, MPS-C was administered from week 3, when the sweating dysfunction was relatively mild, leading us to believe that only the preventive effects of MPS-C on sweating dysfunction have been evaluated. In the future, it is considered necessary to investigate the therapeutic efficacy of MPS-C in STZ-DM mice with advanced autonomic neuropathy caused by diabetes mellitus and severely diminished sweating function, namely, after week 5 as per this study.

In conclusion, these results collectively suggest that MPS prevents reduction in the SC water content by mitigating sweating function impairment through autonomic nerve protection against dry skin observed in STZ-DM mice. These findings suggest that MPS has suitable effects for the treatment of asteatosis induced by sweating function impairment in patients with diabetes mellitus. We will further investigate the detailed mechanism of MPS and demonstrate the peripheral nerve protective effects of MPS in clinical setting in the future to validate the utility of MPS as a therapeutic agent for asteatosis in patients with diabetes mellitus.

## Acknowledgments

We thank Tomo Sasaki and Riichiro Yonezawa for proofreading the manuscript.

## Statement of Ethics

All experimental protocols were approved by the Animal Care Committee of Maruho Co., Ltd. (approval Nos. 22-072, 22-108, 23-014, 23-090, 24-033, 24-060, 25-001, and 25-001A1).

## Conflict of Interest Statement

The authors have no conflict of interest to declare and are employees of Maruho Co., Ltd.

## Funding Sources

This work was supported by Maruho Co., Ltd., and did not receive any specific grant from funding agencies in the public, commercial, or not-for-profit sectors.

## Author Contributions

Wataru Sugimoto: investigation, formal analysis, visualization, conceptualization, and writing – original draft. Hironobu Ishimaru and Sanae Hirota: investigation and writing – review and editing. Ryo Tadakuma, Rina Furumoto, and Ryo Aikawa: investigation and formal analysis. Takaaki Doi: supervision, conceptualization, project administration, and writing – review and editing. Yuhki Ueda: writing – review and editing.

## Data Availability

All data generated or analyzed during this study are included in this article and its supplementary material files. Further inquiries can be directed to the corresponding author.
